# Draft genome sequence and annotations of the mastitis-associated multidrug-resistant *Staphylococcus aureus* strain MBBL_MP1

**DOI:** 10.1128/mra.00073-26

**Published:** 2026-03-12

**Authors:** Kh. Yeashir Arafat, Md. Morshedur Rahman, Sayeda Fowzia Homa, Md. Anamul Kabir Hemal, Md. Samiul Islam Shanto, Mahmuda Nasrin Juthi, Ziban Chandra Das, M. Nazmul Hoque

**Affiliations:** 1 Molecular Biology and Bioinformatics Laboratory, Department of Gynecology, Obstetrics and Reproductive Health, Gazipur Agricultural University 198780, Gazipur, Bangladesh; Fluxus Inc., Sunnyvale, California, USA

**Keywords:** *Staphylococcus aureus*, clinical mastitis, dairy cow, milk, multidrug resistance, virulence

## Abstract

We present the draft genome of multidrug-resistant *Staphylococcus aureus* MBBL_MP1 isolated from bovine clinical mastitis (CM) milk. The 2.75 Mb genome (32.5% GC; 153.4× coverage) contains 29 antimicrobial resistance genes (ARGs) and 65 virulence factor genes (VFGs), highlighting high pathogenic potential.

## ANNOUNCEMENT

*Staphylococcus aureus* is a major mastitis pathogen in cows and buffaloes (1–3) and is frequently isolated with MDR and virulence traits, increasing treatment failure and public health (4–6). Owing to its zoonotic potential and rapid genetic evolution (7), ongoing surveillance is essential. Here, we report the draft genome sequence of an MDR *S. aureus* strain (MBBL_MP1) isolated from milk of a crossbred cow diagnosed with CM (8) in Gazipur (24.09° N, 90.41° E), Bangladesh.

Approximately 10 mL CM milk was collected from the selected cow during morning milking (08:00 h, GMT +6) on 20 March 2025 following standard procedures (9, 10) and transported to the laboratory at 4°C; 100 μL milk was inoculated into 5 mL nutrient broth (Biolife, Italy) and incubated overnight at 37°C under static conditions (11, 12). In total, 2 μL enriched broth was streaked onto Mannitol Salt Agar (Oxoid, UK) and incubated at 37 °C for 24 h. Presumptive *S. aureus* colonies were repeatedly subcultured for purification. Phenotypic identification was based on colony morphology (golden-yellow pigment), Gram staining (gram-positive cocci), and biochemical assays (catalase- and coagulase-positive) (13, 14). Species-level identification was performed using the VITEK-2 system v9.01 (15). A pure colony was suspended in saline, adjusted to the required McFarland turbidity, and processed in a VITEK 2 GP card for species identification using the system’s reference database. The MBBL_MP1 isolate was found to be MDR (resistant to >5 antibiotics), as determined by disk diffusion methods according to the CLSI 2023 guidelines (16). Genomic DNA was extracted from pure colonies of MBBL_MP1 cultured on nutrient agar at 37°C for 24 h under static incubation conditions using the QIAamp DNA Mini Kit (QIAGEN, Germany) (17, 18). DNA purity was evaluated using a NanoDrop 2000 spectrophotometer (A260/A280 ratio) (19, 20), and concentration was determined with a Qubit 4.0 fluorometer (Thermo Fisher Scientific, USA). Whole-genome libraries were constructed from 1 ng of DNA using the Nextera DNA Flex Kit (Illumina, USA), and sequencing was performed on the Illumina MiSeq platform with a 2 × 250 bp paired-end protocol (17). Base calling and demultiplexing were performed using Illumina’s bcl2fastq (v2.20) pipeline to generate paired-end FASTQ files. Raw reads were assessed using FastQC v0.11.3 (21), and Illumina adapters, artifacts, and phiX reads were removed through Trimmomatic v0.39.2 (22). Reads with phred scores >30 were assembled using SPAdes v4.0.0 (23) and annotated through NCBI Prokaryotic Genome Annotation Pipeline v.6.10 (24). Genome completeness was estimated with CheckM v1.2.3 (25). ARGs and VFGs in *S. aureus* MBBL_MP1 were detected using AMRFinderPlus v4. 0.23 (26) and VFDB v6.0 (27), respectively, bundled in ABRicate pipeline (https://github.com/tseemann/abricate). CRISPR arrays and functional subsystems were identified by CRISPRCasFinder v4.2.20 (28) and RAST v2.0 server (29), respectively. The potential pathogenic risk to humans was predicted by PathogenFinder v1.1 (30). Average nucleotide identity (ANI) analysis using ANI Clustermap v2.0.1 (31) revealed >98.0% nucleotide identity with 20 reference *S. aureus* strains retrieved from the NCBI. Default parameters were used unless otherwise specified. The key genomic features of *S. aureus* MBBL_MP1 are presented in Table 1.

**TABLE 1:** Genomic features of *S. aureus* MBBL_MP1 isolated from bovine clinical mastitis milk

Genomic features		*S. aureus* MBBL_MP1
GenBank accession		JBSCUB000000000
BioSample accession		SAMN53123438
Assembled genome size (bp)		2,754,984
Total number of reads (spots)		1,688,616
Total sequence data (Mbp)		450.7
High quality bases (Mbp)		142.8
GC content (%)		32.5
Genome coverage (x)		153.4
N_50_ value (bp)		176,387
Number of contigs		100
Number of scaffolds		44
Genes (total)		2,798
CDSs (total)		2,724
Genes (coding)		2,645
CDSs (with protein)		2,645
Genes (RNA)		74
rRNAs		4, 5, 5 (5S, 16S, 23S)
Complete rRNAs		4 (5S)
Partial rRNAs		5, 5 (16S, 23S)
tRNAs		56
ncRNAs		4
Pseudo genes (total)		79
CDSs (without protein)		79
Pseudo genes (ambiguous residues)		0 of 79
Pseudo genes (frameshifted)		43 of 79
Pseudo genes (incomplete)		32 of 79
Pseudo genes (internal stop)		22 of 79
Pseudo genes (multiple problems)		17 of 79
CRISPR arrays		7
Number of ARGs		29
Number of VFGs		65
Number of functional subsystems		270
Pathogenicity score		0.981
Pathogenic families		466
Phenotypic antimicrobial resistance (zone of inhibition in mm)	Nalidixic acid	11
	Oxacillin	0
	Ampicillin	0
	Nitrofurantoin	13
	Gentamicin	12
	Tetracycline	12

## Data Availability

The whole genome sequence has been deposited in the NCBI/GenBank under BioProject accession number PRJNA1358723, and the NCBI Sequence Read Archive (SRA) accession number SRR35976015. Genome accession numbers are available in Table 1.
